# *Yersinia pestis* BipA is a novel regulator of pesticin and a type 6 secretion system

**DOI:** 10.1128/iai.00191-25

**Published:** 2025-08-11

**Authors:** Madeleine G. Scott, Wanfeng Guo, Jon S. Blevins, Kenneth T. Appell, Roger D. Pechous

**Affiliations:** 1 Department of Microbiology and Immunology, University of Arkansas for Medical Sciences 12215, Little Rock, Arkansas, USA; 2 Microbiology and Immunology, East Carolina University, Greenville, North Carolina, USA; University of California San Diego School of Medicine, La Jolla, California, USA

**Keywords:** BipA, translational GTPase, type 6 secretion, bacterial competition, *Yersinia*, plague, *Yersinia pestis*

## Abstract

*Yersinia pestis* is a gram-negative bacterium and the causative agent of bubonic, septicemic, and pneumonic plague. *Y. pestis* is most commonly transmitted to humans by infected fleas that deposit the bacteria into the dermis at the bite site, leading to bubonic plague. The bacteria ultimately access the bloodstream, and after deposition in the lung, can be transmitted person-to-person through infectious respiratory droplets, resulting in primary pneumonic plague, a highly lethal and rapidly progressing pneumonia. Pathogenesis is mediated by a suite of chromosomally encoded and plasmid-borne virulence factors, and infection is maintained by temperature-dependent coordinated modifications in gene expression that facilitate bacterial survival in both the flea and mammalian hosts. BipA (BPI-inducible protein A) is a highly conserved translational GTPase that is a *Y. pestis* virulence factor. BipA modulates protein expression under stress conditions, and its deletion renders *Y. pestis* more sensitive to killing by neutrophils and attenuates bacterial growth in a murine infection model of pneumonic plague. In the work described here, we show that BipA also regulates specific *Y. pestis* proteins at flea/environmental temperatures. We show that BipA is responsible for the induction of a recently described type 6 secretion system (T6SS), as well as the transcriptional regulator RovC. We also show that BipA regulates the production of the known *Y. pestis* bacteriocin pesticin, and that bacteria lacking BipA have a defect in competition not solely attributable to the T6SS or pesticin. Our results show that in addition to its role in the mammalian host, regulation of specific proteins by BipA also likely contributes to bacterial survival during the flea/environmental phase, where *Y. pestis* must compete with other species of bacteria within a particular niche.

## INTRODUCTION

*Yersinia pestis* is a gram-negative bacillus and the causative agent of bubonic, septicemic, and pneumonic plague. Pneumonic plague results from pulmonary infection with *Y. pestis* and is the most severe manifestation of plague, with 100% mortality if left untreated (1). Pneumonic plague is characterized by a distinct biphasic progression of the disease, with an initial asymptomatic proliferative period (pre-inflammatory phase) followed by the rapid onset of severe pulmonary inflammation (pro-inflammatory phase) that can be fatal within 48–72 h of symptom onset (2). In addition to mammalian hosts, plague maintains an arthropod niche where a suite of differentially regulated genes contributes to colonization of the flea and subsequent transmission to mammalian hosts (3). After a flea feeds on an infected animal, the bacteria form a biofilm on the flea’s proventriculus, a spiny structure between the esophagus and the midgut that facilitates the lysis of red blood cells. Growth of the biofilm results in partial or full blockage of the flea midgut, inducing starvation that results in increased attempts to feed (4–6). Bacteria are then transmitted to the next mammalian host through the dermal bite site. The temperature shift between flea and mammalian hosts is an important environmental signal that directs bacterial gene expression (7) and is modeled in the lab by growing cultures at 26°C to mimic the flea vector and 37°C to mimic the mammalian stage of infection.

Bacterial competition is an important part of the lifecycle for many pathogens, particularly in organisms that have an environmental phase where they compete with bacteria of varying fitness for limited nutrients within the same niche. In some gram-negative bacteria, type six secretion systems (T6SSs) contribute to interbacterial competition by serving as a contact-dependent needle to deliver effector proteins and toxins into prey bacterial cells (8). T6SS has only been recently described in *Yersiniae*, but the conservation of 4–6 T6SS loci in *Y. pestis* and its most recent ancestor *Yersinia pseudotuberculosis* suggests distinct functions for each gene cluster (9). *Y. pestis* also expresses the gene encoding the bacteriocin pesticin (Pst), a known mediator of *Yersiniae* interbacterial killing, on the plasmid pPCP1, which was acquired during its evolution from *Y. pseudotuberculosis*. Pst is a muramidase released from *Y. pestis* that binds to the FyuA receptor on target bacteria. Pst is then transported into the target cell periplasm, where it hydrolyzes peptidoglycan to disrupt the cell wall and cause osmotic stress (10–16). In addition to *pst,* pPCP1 encodes the cognate Pst immunity protein (Pim), which localizes to the bacterial periplasm (17). All *Yersiniae* and many other gram-negative bacteria express the Pst receptor FyuA, rendering them susceptible to Pst-mediated killing (18). However, *Y. pestis* harboring pPCP1 express Pim and are thus resistant to Pst bactericidal activity, preventing self-killing and allowing for selection against other gram-negative bacteria or *Y. pestis* that have lost pPCP1 (19, 20).

BipA (BPI-inducible protein A) is a *Y. pestis* translational GTPase that associates with the ribosome in response to stress signals, including ppGpp, membrane perturbation, and low temperature, resulting in translation of specific proteins (21). BipA is highly conserved among plants as well as several gram-negative bacteria and regulates the translation of virulence-related surface proteins via an unknown mechanism (22–24). Although the BipA regulon has not yet been fully characterized in *Y. pestis*, in other pathogenic bacteria, these proteins include lipopolysaccharide and capsule components, flagellar machinery, and type 3 secretion systems (T3SS) (25–28). BipA was first found to be expressed by *Salmonella enterica* in response to bactericidal permeability-increasing protein (BPI), an antimicrobial peptide produced by granulocytes (29). In addition to *S. enterica* serovar Typhimurium, BipA is present in *Escherichia coli* and *Pseudomonas aeruginosa,* where it controls the cold shock response, biofilm formation, and T3SS components (27, 28, 30, 31). Previous work from our lab established BipA as a *Y. pestis* virulence factor that is important for survival during initial colonization of the lung during pneumonic plague (32). In this study, we show that BipA also regulates the expression of specific proteins at flea/environmental temperature (26°C). We establish BipA as a novel regulator of components of a recently characterized T6SS and Pst, and we show that *Y. pestis* lacking BipA is a less fit competitor during bacterial growth *in vitro*. We predict that BipA plays distinct roles in bacterial survival at both 26°C and 37°C, highlighting its importance during both mammalian and flea/environmental phases of the *Y. pestis* lifecycle.

## RESULTS

### BipA regulates the expression of a *Y. pestis* T6SS

**Fig 1:**
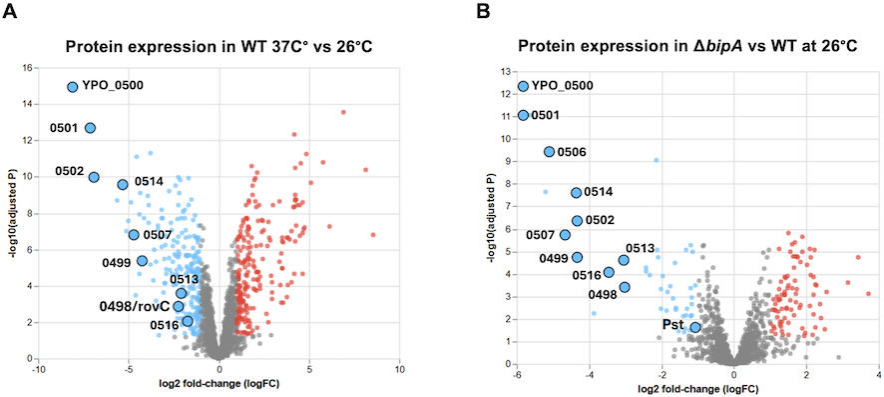
BipA regulates the production of a T6SS at 26°C. (**A**) CFUs (5 × 10^8^) of overnight cultures of CO92 *pgm-*WT *Y. pestis* grown at 26°C or 37°C were submitted to the IDeA National Resource for Quantitative Proteomics for analysis via mass spectrometry and identification of differentially expressed (DE) proteins. Proteins in blue are downregulated at 37°C relative to 26°C. Components of the T6SS cluster A are labeled. (**B**) CFUs (5 × 10^8^) of an overnight culture of WT *Y. pestis* or *ΔbipA Y. pestis* grown at 26°C were analyzed for DE proteins. Proteins in blue are downregulated in *ΔbipA* vs WT, and components of the *Y. pestis* T6SS-A, as well as Pst, are labeled.

Our previous work indicated that BipA regulated the expression of 28 proteins at 37°C in the presence of its inducer BPI (32). Of the most dramatically altered in the absence of BipA were five proteins encoded from a single genetic locus, *YPO0499-YPO516*. This locus is nearly identical to that encoding a T6SS called T6SS4 in the *Y. pestis* ancestor *Y. pseudotuberculosis* (33). T6SS4 is required for resistance to oxidative, low pH, and osmotic stress (34) and is preferentially expressed at 26°C. In 2009, Robinson et al*.* (35) designated this same T6SS cluster in *Y. pestis* as T6SS cluster A (T6SS-A), one of 4–6 putative T6SS loci in the *Y. pestis* genome. While its deletion did not significantly impact the survival of infected mice, T6SS-A was preferentially expressed at 26°C compared to 37°C, suggesting a putative role at flea/environmental temperatures. We, therefore, sought to determine if BipA controls T6SS-A production in *Y. pestis* at 26°C. We compared the proteomes of wild-type (WT) and *ΔbipA Y. pestis* grown overnight at 26°C or 37°C using liquid chromatography-tandem mass spectrometry. This analysis confirmed that components of T6SS-A are preferentially expressed at 26°C compared to 37°C in WT bacteria (Fig. 1A). We observed a number of differentially expressed proteins (95 upregulated and 64 downregulated; Table 1) in *ΔbipA* compared to WT bacteria grown at 26°C, indicating that BipA may be an important regulator of translation outside the mammalian host. Of the 20 most highly downregulated proteins in *ΔbipA*, 9 were found within the T6SS-A locus (YPO499-YPO516), confirming that BipA is largely responsible for the induction of T6SS-A expression at 26°C (Fig. 1B; Table 1). These results identify BipA as a regulator of T6SS production for the first time.

**TABLE 1:** Top 20 upregulated proteins in *ΔbipA* relative to WT CO92 *pgm*− at 26°C and top 20 downregulated proteins in *ΔbipA* relative to WT CO92 *pgm−* at 26°C^*a*^

Upregulated proteins			Downregulated proteins		
Gene ID	Description	Log fold change	Gene ID	Description	Log fold change
YPO0130	Putative exported protein	3.316267439	YPO0500	Uncharacterized protein (TssB)	−5.78617394
YPO1567	L-fuconate dehydratase	3.188191814	YPO0501	Uncharacterized protein (TssC)	−5.7859184
YPO1573	Putative polysaccharide deacetylase	2.987983949	bipA	50S ribosomal subunit assembly factor BipA	−5.535357213
uspA	Universal stress protein A	2.933549346	clpB	Putative Clp ATPase (YPO0506)	−5.308575595
ydjJ	Putative zinc-binding dehydrogenase	2.556795448	YPO0507	Uncharacterized protein (VgrG)	−5.203213111
YPO3648	Putative 2-hydroxy-3-oxopropionate reductase	2.552812958	YPO0499	Uncharacterized protein (TssA)	−4.931923248
YPO2238	Epimerase domain-containing protein	2.513637547	YPO0514	Putative OmpA-family membrane protein (TssL)	−4.882450558
YPO3649	Putative gamma carboxymuconolactone decarboxylase	2.401546915	YPO0502	Uncharacterized protein (Hcp)	−4.424447708
aceK	Isocitrate dehydrogenase kinase/phosphatase	2.397234908	YPO0102	Putative exported protein	−4.238492678
bfr	Bacterioferritin	2.387190803	YPO0513	Uncharacterized protein (TssJ)	−4.143596827
YPO1201	Putative amino acid decarboxylase	2.37034982	YPO0516	Uncharacterized protein (TssM)	−3.844747583
yebF	Protein YebF	2.357816145	YPO3682	Putative lysR-family transcriptional regulatory protein	−3.762334465
dhaK	Putative dihydroxyacetone kinase	2.325708797	cdh	CDP-diacylglycerol pyrophosphatase	−3.181951623
YPO3472	Putative sugar binding protein	2.323643621	YPO0498	DUF2285 domain-containing protein (RovC)	−3.028031982
YPO3349	Uncharacterized protein	2.320414171	b2796	Serine transporter	−2.85239565
YPO2152	UPF0229 protein YPO2152/y2169/YP_1953	2.30350839	YPO3706	Uncharacterized protein	−2.403981039
YPO0904	Uncharacterized protein	2.147001315	YPO3707	Uncharacterized protein	−2.402611965
YPO2202	Putative lipoprotein	2.141492035	YPMT1.66c	Putative DNA-binding protein	−2.364359859
hpaB	4-hydroxyphenylacetate 3-monooxygenase	2.122507199	yiaF	Putative exported protein	−2.263071718
mgtB	Magnesium-transporting ATPase, P-type 1	2.108447492	YPO0639	Uncharacterized protein	−2.116171544

^*a*^ Proteins were sorted by *P*-value (significance: *P* < 0.05) and then by log fold change to generate the top and bottom 20 proteins.

### BipA regulates the expression of the transcriptional regulator RovC

**Fig 2:**
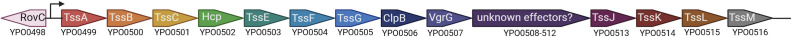
Putative organization of T6SS-A in *Y. pestis*. T6SS-A locus (*YPO0498-0516*) is depicted based on identified sequence similarity to known T6SS loci in *Y. pseudotuberculosis* and *E. coli*. Genes encoded in *YPO0508-0512* have no homology to any currently described proteins.

**Fig 3:**
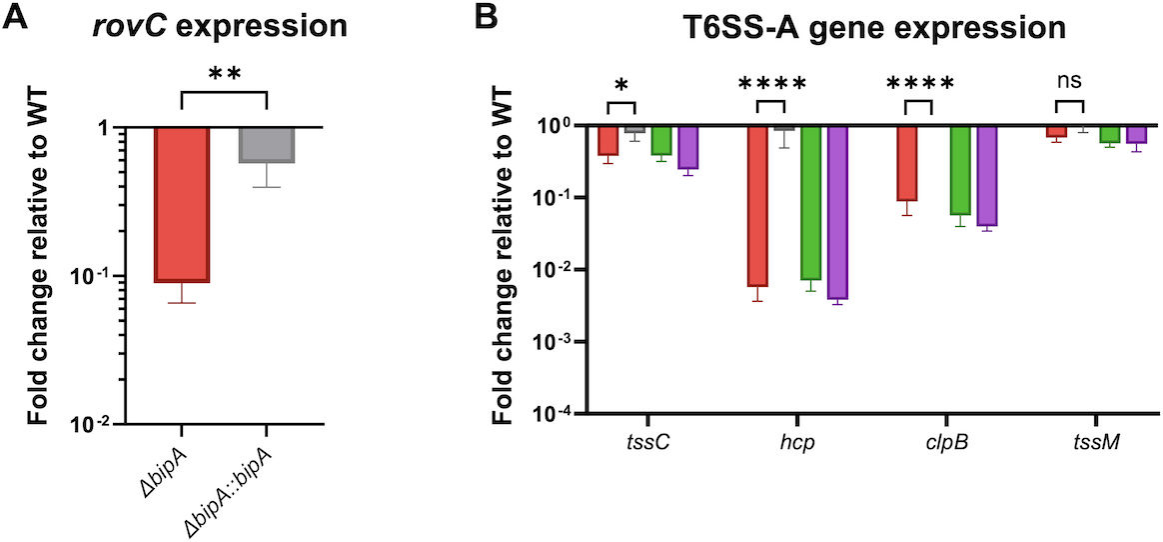
BipA and RovC regulate the production of a T6SS. (**A**) Expression of the indicated T6SS-A genes in *ΔbipA, ΔbipA::bipA, ΔrovC,* or *ΔbipAΔrovC* strains relative to WT *pgm− Y. pestis* after 3 h growth in BHI media. (**B**) Expression of *rovC* in *ΔbipA* or *ΔbipA::bipA* compared to WT *pgm*− after 3-h growth in BHI media. Expression of each gene was normalized to *gyrB* expression for each sample. SEM is shown; significance was calculated (**A**) via two-way ANOVA and Tukey’s multiple comparison or (**B**) via Student’s unpaired *t*-test; ns, not significant; *P ≤ 0.05; **P ≤ 0.005; and ****P ≤ 0.0001.

**Fig 4:**
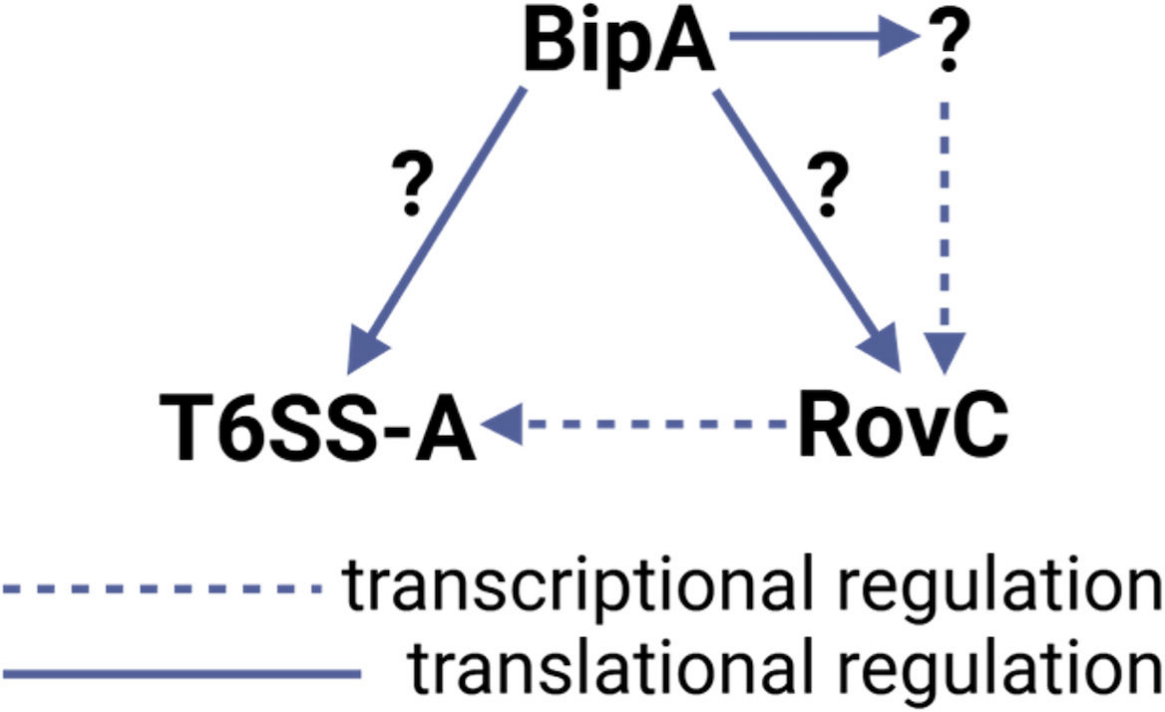
BipA-mediated regulation of T6SS-A. BipA regulation of RovC results in increased transcription of T6SS-A genes. While BipA may induce T6SS-A and/or *rovC* translation, BipA also impacts the expression of a yet-to-be-identified activator of RovC transcription.

The absence of BipA also saw dramatically reduced expression of YPO0498, a protein with unknown function, with an open reading frame immediately adjacent to the *YPO0499-YPO0516* locus oriented in the opposite direction. An NCBI BLAST search of YPO0498 showed 100% sequence homology to Regulator of Virulence C (RovC), recently described in *Y. pseudotuberculosis* as a transcriptional regulator of T6SS4. Under specific environmental conditions, RovC activates transcription of T6SS4 to import and export ions and other products to respond to various stressors. Figure 2 shows the organization of the *Y. pestis* T6SS-A locus based on homology with known T6SS clusters from *Y. pseudotuberculosis* (9, 36) and *E. coli* (37). The finding that deletion of BipA resulted in decreased RovC expression indicates that BipA-mediated induction of T6SS-A may result from direct translational regulation or indirectly through the impact of BipA on the expression of the transcriptional activator RovC. To examine the regulation of RovC and T6SS-A in greater detail, we measured the expression of *rovC* and T6SS-A genes in WT, *ΔbipA, ΔrovC,* and *ΔbipAΔrovC Y. pestis* using quantitative real-time PCR (qRT-PCR). Since the loss of *bipA* unevenly impacted the translation of the T6SS-A components in our proteomic analyses, we selected several genes essential to the T6S apparatus that span the entire locus for further analysis. The tail complex protein TssC (*YPO0501*), Hcp (*YPO0502*), and the inner membrane protein TssM (*YPO0516*) are critical structural components of T6SS-A, while the clB ATPase (*YPO0506*) controls assembly and release of cargo. By utilizing qRT-PCR in concert with proteomic analysis, we can determine if gene expression matches protein production and thereby better assess if BipA regulates T6SS-A directly or indirectly through RovC. We predicted that direct translational regulation of the T6SS-A locus by BipA would see no difference in gene expression in *ΔbipA* compared to WT bacteria, whereas indirect regulation through RovC or other transcriptional regulators would see differential expression of genes within the T6SS-A locus in the absence of BipA. First, we attempted to confirm that BipA regulates RovC at the translational level, but we were surprised to find that *rovC* expression was significantly reduced in *ΔbipA* compared to wild-type bacteria (Fig. 3A). While regulation at the translational level may still occur, this indicates that BipA-dependent regulation of RovC is also indirect, likely through the impact of BipA on an upstream mediator of *rovC* transcription. Alternatively, RovC may positively regulate its own transcription, as has been shown in *Y. pseudotuberculosis*. In a similar trend to *rovC*, we observed reduced expression of most T6SS-A genes in *ΔbipA* relative to WT or *ΔbipA* complemented with wild-type *bipA* (Fig. 3B), also suggesting that regulation of T6SS-A by BipA is indirect, or not solely at the translational level. Surprisingly, not all genes within the locus were equally impacted by the loss of *bipA*, implying that there may be additional regulators impacting the genes of this locus. The decrease in the expression of T6SS-A genes in *ΔbipAΔrovC* was equivalent to what was seen in *ΔrovC* and *ΔbipA*, indicating that BipA regulation of RovC is likely responsible for expression of T6SS-A. These results indicate that BipA regulation of T6SS-A is complex and likely involves the regulation of expression of transcriptional regulators as intermediates (Fig. 4).

**Fig 5:**
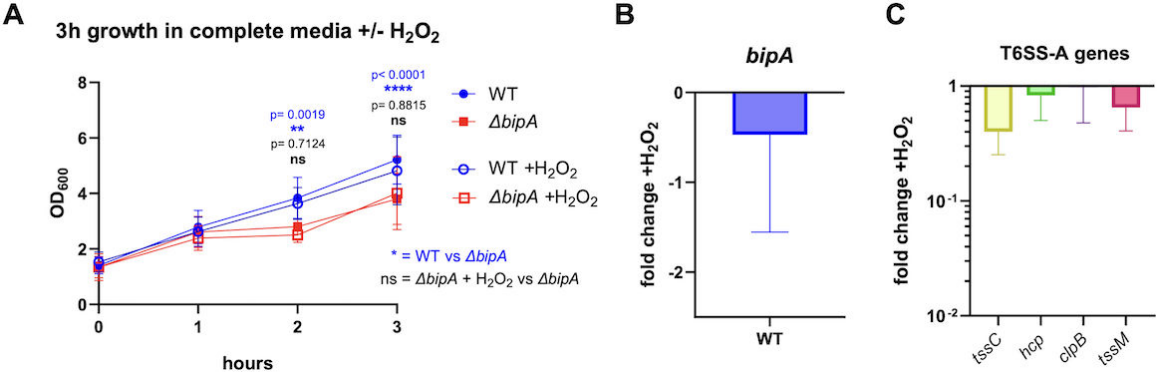
*Y. pestis* T6SS-A and BipA are not induced by oxidative stress at 26°C. (**A**) Measurement of cultures at OD_600_ after 1.5 mM H_2_O_2_ challenge in BHI complete media. (**B**) Expression of *bipA* in WT *pgm*− after 3-h growth in PMH minimal media with 1.5 mM H_2_O_2_ relative to *bipA* expression without 1.5 mM H_2_O_2_. (**C**) Fold change in the relative expression of T6SS-A genes in WT *pgm*− after 3-h growth in PMH minimal media with 1.5 mM H_2_O_2_ relative to their expression without 1.5 mM H_2_O_2_. Significance in panel A was determined by two-way ANOVA and Tukey’s multiple comparison test between all growth conditions and backgrounds at each time point. Blue text shows the significance of WT vs *ΔbipA Y. pestis* in the absence of H_2_O_2_, while black text shows the comparison of *ΔbipA* in the absence vs the presence of H_2_O_2_. In panel C, “induction” with H_2_O_2_ did not result in changes in gene expression, and thus no statistical analysis was performed.

Hydrogen peroxide (H_2_O_2_) is a known inducer of T6SS expression, including the T6SS-A homolog in *Y. pseudotuberculosis* T6SS4 (38–40), and T6SS4 contributes to *Y. pseudotuberculosis* survival under oxidative stress conditions (39). Additionally, BipA is known to respond to oxidative stress (23, 24, 30, 41, 42). We therefore sought to determine if BipA regulates T6SS-A components in the presence of H_2_O_2_ in *Y. pestis*, and if BipA-mediated regulation of T6SS-A contributes to bacterial survival under oxidative stress. Addition of a sublethal concentration of H_2_O_2_ to actively growing cultures resulted in slightly reduced growth of both WT and *ΔbipA* strains of *Y. pestis* (Fig. 5A). The absence of BipA, though, did not impact the growth of *Y. pestis* in the presence of H_2_O_2_ compared to WT bacteria, indicating that BipA regulation of T6SS-A does not contribute to resistance to oxidative stress. Unlike what is observed in *Y. pseudotuberculosis*, the addition of 1.5 mM H_2_O_2_ to cultures did not impact the expression of T6SS-A genes and also had no impact on *bipA* transcription (Fig. 5B and C). These results indicate that, unlike in *Y. pseudotuberculosis*, T6SS-A does not contribute to *Y. pestis* survival under oxidative stress conditions at 26°C.

### BipA controls Pst production

**Fig 6:**
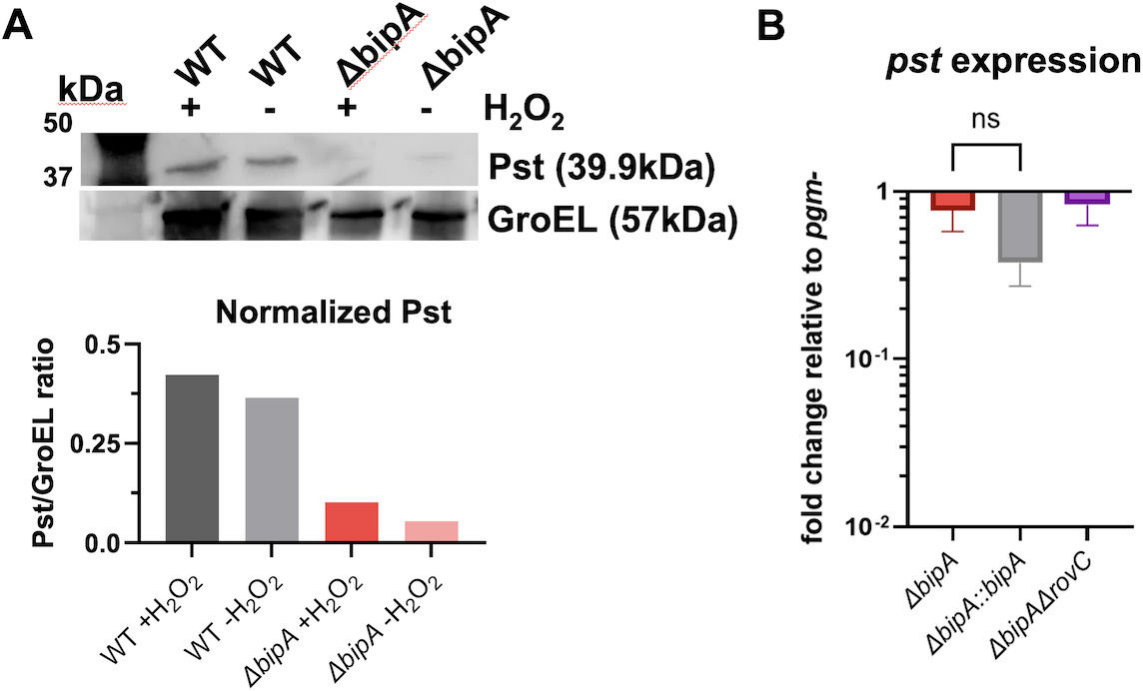
BipA regulates Pst production at the translational level. (**A**) Equal concentrations of protein lysates from *pgm*− WT or *ΔbipA Y. pestis* strains grown at 26°C were run on a 10% Tris-glycine gel before transfer to a nitrocellulose membrane and blotting with 1:1,000 Pst-specific polyclonal rat sera. The membrane was stripped and reprobed with a monoclonal antibody to GroEL as a loading control. Pst levels were normalized to GroEL using densitometry analysis and ImageJ. (**B**) Relative expression of *pst* in *ΔbipA, ΔbipA::bipA,* or *ΔbipAΔrovC* compared to relative expression in WT *pgm*− after 3-h growth in BHI complete media. Significance in panel B was determined by two-way ANOVA and Tukey’s multiple comparison test between all background strains.

Our proteomic analysis also implicated BipA as a regulator of Pst for the first time (Fig. 2B). Pesticin is the canonical *Y. pestis* bacteriocin that hydrolyzes peptidoglycan, causing osmotic stress and eventual cell lysis of target bacterial cells (10–12, 14, 16, 43, 44). To confirm that BipA is a novel regulator of Pst, we performed Western blot analysis on protein lysates from WT and *ΔbipA* bacteria using polyclonal rat antisera specific for Pst (~39 kDa). We observed decreased levels of Pst in *ΔbipA* relative to WT bacteria (Fig. 6A), supporting our proteomics data indicating that BipA induces Pst production. As we initially hypothesized that BipA regulated proteins under oxidative stress conditions, we also examined Pst expression in cultures grown in the presence of H_2_O_2_. Similar to what was seen for T6SS-A expression, the presence of H_2_O_2_ did not appear to impact BipA-mediated induction of Pst expression. To determine if this regulation also occurs at the transcriptional level, we performed qRT-PCR on mid-log phase culture to measure the expression of *pst* in *ΔbipA*. The absence of BipA did not significantly impact the transcription of *pst*, indicating that the regulation of Pst by BipA is likely at the translational level (Fig. 6B). These results implicate BipA as a novel translational regulator of Pst production and may have implications for bacterial survival during competition with other bacteria.

### BipA contributes to interbacterial competition in *Y. pestis*

**Fig 7:**
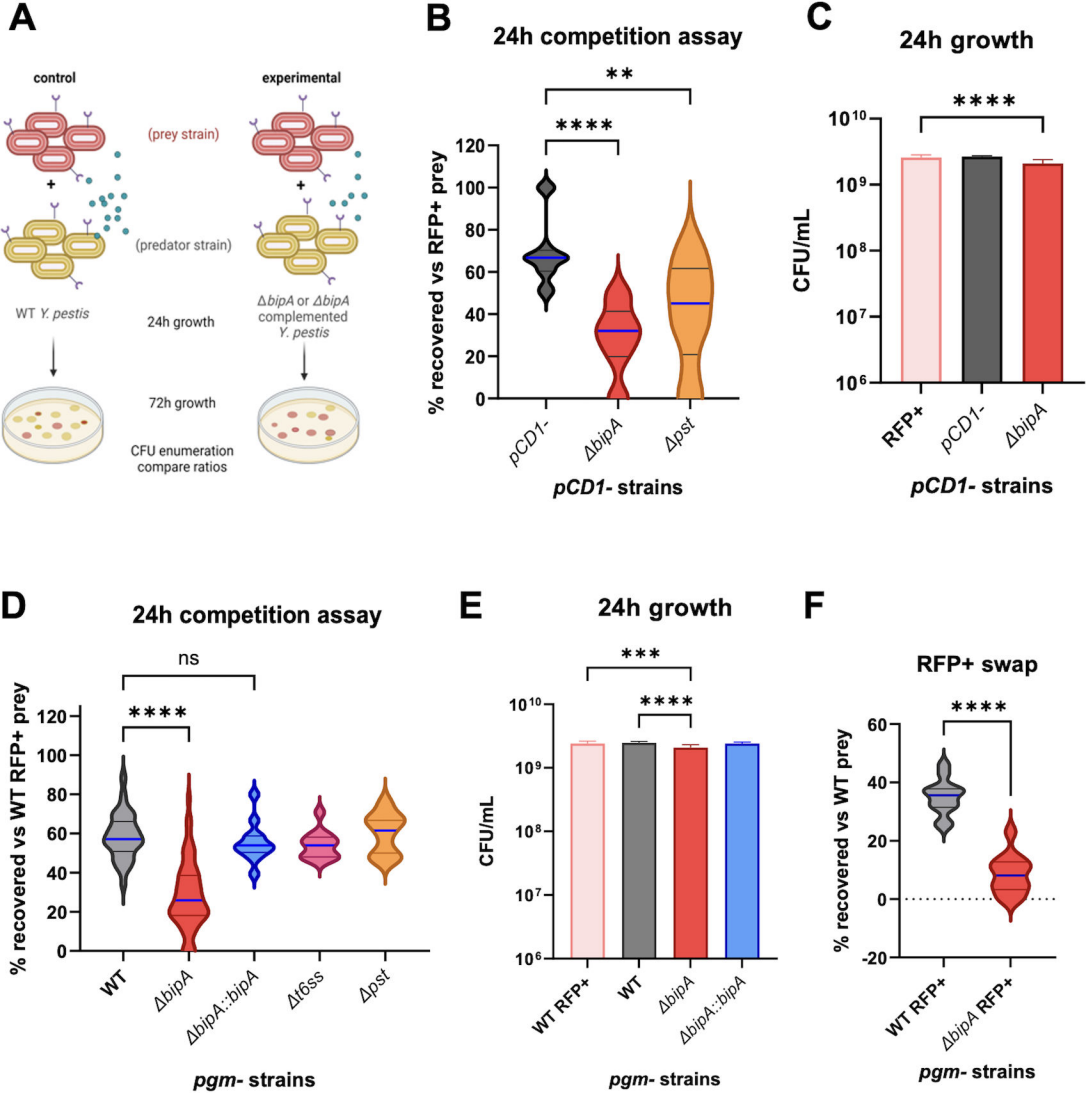
BipA impacts bacterial competition in *Y. pestis*. (**A**) Schematic showing competition assay setup, with RFP+ prey used for colony enumeration. (**B**) CO92 *pCD1*− 24-h coculture competition assays in BHI complete media. (**C**) *pCD1*− strains single culture CFU after 24-h growth in 2 mL of BHI. (**D**) CO92 *pgm*− WT 24-h competition assays in BHI media. (**E**) *pgm*− WT strains single culture CFU after 24-h growth in 2 mL of BHI media. (**F**) *pgm*− WT 24-h competition assays, with RFP+ predator used for colony enumeration. (**B–E**) Significance was determined by two-way ANOVA. (**F**) Significance determined by Student’s *t*-test; *P ≤ 0.05, **P ≤ 0.005, ***P ≤ 0.0005, and ****P ≤ 0.0001.

Pst is a known mediator of bacterial killing (12, 15, 43), and T6SS is often associated with interbacterial competition and toxin production (8, 45–48). The decreased production of at least two molecules implicated in bacterial competition in *ΔbipA* led us to ask if BipA contributes to bacterial survival during interbacterial competition. To test this, WT CO92 pCD1− bacteria harboring a plasmid encoding red fluorescent protein (RFP+ “prey”) were cocultured for 24 h with either WT or *ΔbipA Y. pestis* lacking the plasmid (white colonies), and ratios of red-to-white colonies were determined by standard plate count (Fig. 7A). CO92 pCD1− harbors the pigmentation locus that encodes the Pst receptor FyuA. While this strain also expresses Pim and exhibits some resistance to Pst-mediated killing, we still expect to see a slight decrease in survival for bacteria lacking Pst when competing with wild-type bacteria. When prey and predator bacteria were cultured together, we consistently recovered significantly fewer *ΔbipA* colonies than WT or complemented *ΔbipA::bipA* strains when competed against the same prey, indicating that BipA does contribute to survival during interbacterial competition (Fig. 7B). As expected, *Y. pestis* lacking *pst* also showed reduced survival in coculture, confirming a likely role for Pst during interbacterial competition in our assay. When pCD1− strains were cultured separately in brain heart infusion (BHI) at 26°C, we did observe a small growth defect in *ΔbipA* (Fig. 7C), but this is not enough to account for the differences in recovery post-competition. Of note, while not statistically significant, the deletion of BipA appeared to impart a more dramatic growth defect compared to the absence of Pst in coculture, indicating that BipA may regulate elements in addition to Pst that contribute to interbacterial competition.

As our data indicate that BipA may have a greater role in our assay than Pst alone, we sought to determine if BipA impacted interbacterial competition independent of Pst. To this end, we repeated coculture assays with WT *Y. pestis* lacking the *pgm* locus (*pgm*−). The *pgm* locus harbors the Pst receptor Fyu; thus, the absence of the *pgm* locus excludes a role for Pst from the assay. As expected, the absence of Pst did not impact bacterial competition in coculture with this strain (Fig. 7D). As in the pCD1− strains, the absence of BipA resulted in decreased bacterial fitness, as we recovered fewer *ΔbipA* bacteria after 24 h in coculture (Fig. 7D) than when strains were cultured separately (Fig. 7E). Importantly, we observed this effect regardless of which strain carried the RFP-expressing plasmid (Fig. 7F). These results confirm that BipA contributes to interbacterial competition. As T6SSs contribute to interbacterial competition in *Serratia, Vibrio, Y. pseudotuberculosis,* and some plant pathogens (49–53), we next sought to determine if the defect we saw in *ΔbipA* coculture was due to decreased expression of T6SS-A. To this end, we repeated the competition assay using bacteria lacking the T6SS-A locus (*ΔYPO499-YPO516*). The absence of the T6SS-A locus had no impact on bacterial survival during coculture, suggesting that this locus is not responsible for the *ΔbipA* competition defect (Fig. 7D). These results indicate that BipA regulates proteins that, in addition to Pst and the T6SS-A locus, contribute to *Y. pestis* survival during interbacterial competition.

## DISCUSSION

The *Y. pestis* translational GTPase BipA contributes to virulence during pneumonic plague and promotes bacterial survival in the face of challenges with neutrophils and the antimicrobial BPI. In the work described here, we show that BipA likely also contributes to bacterial survival outside of the mammalian host. In support of this idea, we present several novel findings regarding BipA at flea/environmental temperatures. We show that BipA regulates numerous *Y. pestis* proteins at 26°C, and several of the most dramatically dysregulated proteins in the absence of BipA are components of a recently described *Yersinia* T6SS, which has been shown in *Y. pseudotuberculosis* to be involved in metal ion acquisition, oxidative stress resistance, and membrane stress responses. This work positions BipA as a regulator of T6SS for the first time. Furthermore, we show that BipA induces the expression of the newly discovered transcription factor RovC, shown to be a transcriptional activator of the same T6SS. Additionally, we describe BipA as a regulator of the well-described bacteriocin Pst for the first time. Finally, we show that bacteria lacking BipA are less fit competitors *in vitro* when grown with WT bacteria. Based on these findings, we predict that in response to environmental stressors, BipA induces the expression of proteins that contribute to *Y. pestis* survival during interbacterial competition to aid in the colonization of a particular environmental niche.

Based on the sequence similarity and corresponding phenotype, we have identified RovC for the first time in *Y. pestis* and have shown that RovC is part of the BipA regulon. RovC was recently discovered as a hexameric nucleic acid binding protein in the *Y. pestis* ancestor *Y. pseudotuberculosis*, where it is required for the induction of the T6SS4 locus (36). The authors noted that this regulatory network could fine-tune the expression of T6SS4 and served as a “switch,” facilitating rapid induction of the apparatus based on specific environmental signals. The expression of *Y. pseudotuberculosis rovC* is controlled by the PhoP/PhoQ two-component system through the global regulator Csr, which responds to nutrient/ion availability and low pH to induce transcription of T6SS4 at the stationary phase (36, 54–56). The Csr regulatory system is complex and regulates several cell functions related to virulence and stress responses in response to nutrient and ion availability in the environment. In *Y. pseudotuberculosis*, the regulator CsrA suppresses *rovC* expression under non-inducing conditions, and its derepression is coordinated by environmental signals and controlled by the BarA/UvrY and PhoP/PhoQ two-component regulatory systems (55, 56). In the absence of the repressor CsrA, RovC binds directly to the promoter region upstream of the T6SS operon, exposing the DNA for binding and promoting transcription of both itself and the downstream T6SS genes. Our data suggest that in *Y. pestis,* BipA feeds into or works as a part of this regulatory network to activate the expression of RovC, thereby inducing the expression of the T6SS-A locus. It is yet to be determined if *Y. pestis* BipA is induced by the same stimuli that results in Csr-mediated derepression of *rovC* in *Y. pseudotuberculosis*. To date, it has been shown that BipA regulates the cold shock response and is likely responsive to membrane stress such as that mediated by BPI (22, 57, 58). Work identifying the conditions under which BipA is active is ongoing. Coordinate regulation of transcriptional activators might also explain the mechanism of specificity of translational GTPases. While it is clear that BipA regulates specific surface-associated structures like flagella, pili, and secretion systems, the mechanism of specificity remains unclear. Coordinate or direct induction of transcriptional regulators may introduce a scenario where transcript abundance dictates protein expression, and thus, specificity is not conferred at the translational level. Furthermore, by inducing a transcriptional regulator like RovC, BipA can coordinately regulate the expression of any number of stress response loci, the expression of which BipA may further impact at the translational level.

While T6SS-A is highly induced at 26°C, its precise function and the function of each of the *Y. pestis* T6SS loci remain elusive. In *Y. pseudotuberculosis,* expression of T6SS4 is activated under stress conditions through multiple critical pathways, including the OxyR, OmpR, ZntR, and Csr systems (59–61). T6SS4 is upregulated in response to osmotic stress, oxidative stress, metal ion concentrations, and at 26°C relative to 37°C (39, 59, 62–65), highlighting the putative importance of this locus in general and oxidative stress responses (36, 54, 56, 59, 63). In addition to oxidative stress responses, T6SS4 is required for *Y. pseudotuberculosis* survival in response to high osmolarity and bile salt stress (62). Temperature is an important cue for T6SS assembly, and although previous studies (35, 66) showed the *Y. pestis* T6SS-A locus to be preferentially expressed at 26°C rather than 37°C, a mutant lacking this locus infected *Xenopsylla cheopis* rat fleas to the same level as WT bacteria (35). Curiously, this mutant was also more easily phagocytosed by J774.A1 macrophages, and the authors noted that in late-phase, acidic (pH 5.5), 37°C cultures, the *ΔT6SS-A* mutant reaches a higher optical density than WT (59). The finding that *Y. pestis* lacking BipA did not exhibit a growth defect in the presence of H_2_O_2_ suggests that the role of T6SS-A may differ from its homolog in *Y. pseudotuberculosis* and does not contribute to bacterial resistance to oxidative stress at environmental temperatures. Current work is focused on the role of BipA-mediated T6SS-A expression under various stress conditions.

We have identified BipA as a regulator of Pst for the first time. As indicated by proteomics and confirmed via Western blot analysis, loss of BipA in *Y. pestis* results in decreased Pst production but not *pst* expression as measured by qRT-PCR. Thus, BipA modulates Pst production at the translational level. Despite being the canonical *Y. pestis* bacteriocin, there is still much to learn about Pst. It is still unclear how it is transported out of the cell, and the kinetics of the siderophore yersiniabactin versus Pst affinity for binding to their shared receptor FyuA has yet to be disentangled. As the T6SS is often associated with effector-immunity pairs (67) and can carry diverse cargo (68), and Pst has a cognate immunity protein Pim, it is tempting to speculate that under certain conditions, Pst could be T6SS cargo. In line with this thinking, two recent papers on *Vibrio* species have demonstrated that bacterial competitiveness can be mediated by a “pesticin-like” (69) or a “pesticin domain-containing” (50) periplasmic toxin produced by T6SS, providing an intriguing future target for study. Regardless, the finding that BipA regulates Pst has implications for *Yersinia* competition with other bacteria. Many bacteria, particularly Gammaproteobacteria like *Escherichia coli*, *Klebsiella*, and *Salmonella*, harbor the pigmentation (*pgm*) locus and, therefore, the Pst receptor FyuA (70–76). The ancestral and less pathogenic species *Yersinia enterocolitica* and *Y. pseudotuberculosis* carry the Pst receptor but lack the plasmid pPCP1, and therefore Pim, and are sensitive to Pst-mediated killing (17, 77–81). It is conceivable that after the acquisition of pPCP1, *Y. pseudotuberculosis* clones with the propensity to positively regulate Pst through the effects of BipA gained a selective advantage and, as a result, emerged as a predominant precursor of modern *Y. pestis*, though there is currently no evidence to support this idea. Regardless, the implications of BipA regulation of Pst remain unclear, and future work will focus on elucidating what advantage, if any, is afforded by translational regulation of Pst by BipA in environmental niches such as the flea vector.

Pathogenic bacteria, particularly those that colonize an insect or arthropod vector as part of their life cycle, must compete with other bacteria to occupy a particular niche in the vector and mammalian hosts. For many bacteria, T6SS plays a role in contact-dependent interbacterial competition (82–84), as well as contact-independent targeting of eukaryotic cells (63, 83, 85–88). The absence of BipA saw reduced fitness of *Y. pestis* in coculture with WT bacteria. Pst is a known mediator of interbacterial competition, and the known role of many T6SSs in interbacterial competition in other bacteria led us to test whether the fitness defect resulted from decreased expression of T6SS-A. While BipA regulation of Pst likely contributes to increased survival during competition with other bacteria that carry the FyuA receptor under certain conditions, we found that deletion of either Pst or T6SS-A did not result in competition defects in our assays. These data suggest that a BipA-regulated protein other than members of the T6SS-A locus or Pst contributes to the loss of fitness in *ΔbipA* when incubated with WT bacteria. Our proteomic screen identified two other proteins, which were also downregulated in *ΔbipA*; e.g., MdaB NADPH oxidoreductase and YiaF protein. In *Helicobacter pylori*, MdaB plays a role in oxidative stress resistance, where a *ΔmdaB* mutant cannot colonize the host stomach (89). *E. coli* YiaF is an inner membrane protein that is involved in maintaining bacterial biofilm and resisting threats from competing bacteria (90–92). Interestingly, a transposon mutant screen identified YPO2159, also downregulated in *ΔbipA*, as a unique unidentified protein predicted to aid survival under certain stimuli at 37°C but not at 26°C (93), which could contribute to the role of BipA during pneumonic plague. Current work is focused on identifying mediators of bacterial survival other than Pst that might explain the defect seen during bacterial coculture for *Y. pestis* lacking BipA.

In conclusion, BipA is a translational regulator that likely promotes bacterial survival during all phases of the *Y. pestis* lifecycle, both inside and outside of the mammalian host. We have identified BipA as a regulator of a T6SS for the first time and showed that BipA positively regulates the expression of the hexameric transcriptional regulator RovC. Therefore, it likely impacts the regulation of a number of stress response proteins. Furthermore, BipA is a novel and *bona fide* regulator of mediators of interbacterial competition, including the bacteriocin Pst. We predict that BipA regulates proteins important for bacterial survival and stress responses both inside and outside of the mammalian host.

## MATERIALS AND METHODS

### Bacterial strains

*Y. pestis* strains CO92, CO92 Δ*bipA*, CO92 Δ*pgm*, and CO92 Δ*pCD1* were obtained from the lab of William Goldman (UNC-Chapel Hill). See Table 2 for a complete list of strains. The Δ*bipA Y. pestis* strain was generated using a modified lambda red recombination described previously (94). Upstream and downstream sequences of *bipA* and any other mutants were amplified by PCR using the primers in Table 3. Underlined sequences indicate tags complementary to sequence tags added to primers used to amplify a kanamycin resistance marker. The PCR product of the sequence immediately upstream and downstream of the target loci was then used with the kanamycin resistance cassette as the template for splicing by overlap extension PCR to generate a PCR fragment for allelic exchange. *Y. pestis* strains were grown on BHI agar (Gibco #237300) at 26°C for 2–3 days. BSL-2 mutant strains were confirmed by PCR for the absence of the *pgm* locus or the pCD1− plasmid and the presence of all other virulence factors (Yops and *hms*). Unless otherwise stated, growth curves, competition assays, and Western blots were performed on equal concentrations of bacterial strains subcultured from 2 mL overnight cultures grown in BHI at 26°C in a roller drum. For bacterial growth curves, the optical density (OD_600_) of *Y. pestis* cultured in BHI medium was obtained using a spectrophotometer at the indicated time points.

**TABLE 2:** *Yersinia pestis* strains used in this study

Bacterial strain names	Description
*pgm*− (“WT”)	CO92 *Y. pestis* lacking the pigmentation locus (*pgm*)
*pCD1−*	CO92 *Y. pestis* lacking the pCD1− plasmid
*ΔbipA*	CO92 *pgm*− or *pCD1*− lacking *YPO0026, bipA*
*ΔbipA::bipA*	CO92 *pgm*− lacking *YPO0026* and expressing Tn7+ *bipA*
*ΔrovC*	CO92 *pgm*− lacking *YPO0498*, putative T6SS-A regulator
*Δt6ss*	CO92 *pgm*− or *pCD1*− lacking *YPO0499-0516* (T6SS-A)
*Δpst*	CO92 *pgm*− or *pCD1*− lacking pesticin on the pPCP1 plasmid
*ΔbipAΔrovC*	CO92 *pgm*− lacking *YPO0026* and *YPO0498*
RFP+	CO92 *pgm*− or *pCD1*− expressing pGEN-RFP+ plasmid (amp^R^)
*ΔbipA* RFP+	CO92 *pgm*− or *pCD1*− lacking *YPO0026* and expressing pGEN-RFP+ plasmid (amp^R^)

**TABLE 3:** Primers used in this study.^*a*^

Primer type	Gene name	Direction	Primer sequence
PCR primers for mutagenesis	bipA/YPO0026	5′F	5′-TTA CTG CAT TTA TGG TGT TCA GGC ATT-3′
		5′R	5′-GAA GCA GCT CCA GCC TAC ACC ACA GCT TTT TTG CCT CAG GCA TTT AG-3′
		3′F	5′-GGT CGA CGG ATC CCC GGA ATT TCT TCT TTG CAT TGT TGA TAC TTA GGG C-3′
		3′R	5′-CTG CTC GAA ACT CAA GGA TAT CCC-3′
	kanR cassette	F	5′-GTG TAG GCT GGA GCT GCT TC-3′
	(*E. coli* pKD13)	R	5′-ATT CCG GGG ATC CGT CGA CC-3′
	YPO0502	5′F	5′-GTT TAC CTT ACA TTT TTG CCA CCT G-3′
		5′R	5′-GAA GCA GCT CCA GCC TAC ACC ATT TTT TCA CTC CTG AAT TAA TGT CAT TA-3′
		3′F	5′-GGTCGACGGATCCCCGGAATGTCAGAAATGCATTAGGAC-3′
		3′R	5′-ATTCCATAGGAGTAATCGAGCGCGA-3′
	psaA	5′F	5′GGT CGA CGG ATC CCC GGA ATA TAA CGA GTA ATC ATA GAA CAT GAT GG-3′
		5′R	5′AAT AAA TAT GGC GTC TGT CTA TAT TGG TAT-3′
		3′F	5′GAA GCA GCT CCA GCC TAC ACC ATG AGA ACA GTC TCC ATT AAA TGT AAT AA-3′
		3′R	5′-TGC AGT GTT TCA CGA TCT TCA GGG A-3′
	rovC/YPO0498	5′F	5′ TTA AAT GCC ATT CCA GAT TAT AGG C 3′
		5′R	5′ GAA GCA GCT CCA GCC TAC ACT GTC CTA TCT GAC ACG CAA AGG ATC TAA AA 3′
		3′F	5′ GGT CGA CGG ATC CCC GGA ATA TCT ATG TCC TCT TAT TTT GGC TAT TCA TC 3′
		3′R	5′ GCA GCT TAT TGG CTT GCT CAC TGA T 3′
	pst/pesticin	5′F	5′ GGT TCC ACC CCT TCC GGT TTT TTT C 3′
		5′R	5′ GAA GCA GCT CCA GCC TAC ACC ATA AAA ATT CTT TAA CAC ATA AAA AA 3′
		3′F	5′ GGT CGA CGG ATC CCC GGA ATC CAT GAG CCC CTC ATA ATA ATG AGG GGC TT 3′
		3′R	5′ CCA AAT AAT TTA TTT ATG TAA GAA C 3′
	tssM/YPO0515	5′F	5′ ATG GCT ATT AAT CTT CAC CTC CGA C 3′
		5′R	5′ GAA GCA GCT CCA GCC TAC ACA CAA TAT AAC AAA GCA CTA GGG CAA T 3′
		3′F	5′ GGT CGA CGG ATC CCC GGA ATC CGC GAA ATA AGG AAA 3′
		3′R	5′ CAG TCT GAT ATC CGG GGC A 3′
	pimpst (pp)	5′F	5′ TAA GAC ACG ACT TTA CGC CAC TGG C 3′
		5′R	5′ GAA GCA GCT CCA GCC TAC ACC ATT CCC CCT CCC ATC CTG TTC T 3′
		3′F	5′ GGT CGA CGG ATC CCC GGA ATA AAA ATT CCT CTT TAA CAC ATA AAG AAA AC 3′
		3′R	5′ TCC CGT TCC TGC CCT TCC CGG 3′
	t6ss/YPO0499-0515	5′F	5′ CCC CAT TTT TTT TCT GCA TTC AAA TCC 3′
		5′R	5′ GAA GCA GCT CCA GCC TAC ACC AAA AAT AGC TTG CTA TTC CTT GCT 3′
		3′F	5′ GGT CGA CGG ATC CCC GGA ATC CGC GAA ATA AGG AAA TGC 3′
		3′R	5′ GCA GTC TGA TAT CCG GGG CA 3′
qRT-PCR primers	bipA/YPO0026	F	5′ CGA ACA ACT GGA CTT CCC TAT 3′
		R	5′ GGG TCA TAT CCT CTG CCA TAT 3′
	YPO0502	F	5′ TAG GTG GCG GTG GTG GTT CT 3′
		R	5′ CAC CAA CTT GGC CTG CGG AA 3′
	gyrB	F	5′-ATC CGT TGT TAA CGC CCT GTC TGA-3′
		R	5′-ACA GTA GTC CCG GTT TGC TCA GTT-3′
	phoP	F	5′ TGG CCC ATT TCA CGC ATT TG 3′
		R	5′ TAT GCG GGT TCT GGT TGT GG 3′
	rovC/YPO0498	F	5′ GAG GAA GTT CAG GTA GCC GT 3′
		R	5′ ATG GTC TGC TGA CAG TTG GG 3′
	clpB/YPO0506	F	5′ TGT TGA AGC CAC CCG TCT TT 3′
		R	5′ ATG CTT TGG TGA CGG GCA TA 3′
	tssC/YPO0501	F	5′ ATT TGA AAT GCG GGC GAA CC 3′
		R	5′ CCC ACC AAC GGA TGT TCG TA 3′
	tssM/YPO0515	F	5′ TTG GCC CCT ATT TCC CTC TG 3′
		R	5′ CCA CGG TTT AGC ATC GAT ATA CTG G 3′
	pst/pesticin	F	5′ TTG AAG CTA ATT CGA TTA CAA TTG C 3′
		R	5′ GTT CTC TTT CTT ATC GAG ATG TTC A 3′

^*a*^ Underlined sequence highlights tags for overlap extension PCR.

### Proteomic analysis

To obtain protein extracts from broth-grown *Y. pestis*, 5 × 10^8^ CFU of *Y. pestis* CO92 *pgm*− and *Y. pestis* CO92 *pgm*− Δ*bipA* from overnight cultures were spun down at 10,000 × *g* for 5 min to pellet bacterial cells. Bacterial pellets were resuspended in 1× RIPA buffer (Thermo Scientific #89901) and boiled for 5 min at 96°C to lyse cells. Equal concentrations of each cell lysate sample (approximately 50 ng/µL) were submitted to the IDeA National Resource for Quantitative Proteomics for LC-MS/MS analysis and protein identification and quantification. Data were exported from MaxQuant and in-house bioinformatics analysis software to Microsoft Excel for further statistical analyses.

### Competition assays

Equivalent concentrations from overnight cultures of prey (RFP+ CO92 *pgm*− or *pCD1*− strains) and predator (CO92 *pCD1*− or CO92 *pgm*− Δ*bipA,* Δ*pst,* Δ*tssM,* Δ*t6ss* Δ*rovC,* or Δ*pimpst*) equal to 0.1 OD_600_ were added to 2 mL BHI and spun in a roller drum at 26°C for 24 h. Ten microliters of the coculture was diluted 10^−5^ (*pgm*− cultures) or 10^−6^ (*pCD1*− cultures), and 20 µL was plated on BHI agar for colony enumeration after 2–3 days of growth at 26°C. Data are plotted as number of white colonies/number of total colonies or number of red colonies/number of total colonies (for RFP+ swap experiments) and represent three to four experiments.

### Oxidative stress assays

Equivalent concentrations from overnight cultures of CO92 *pgm−,* Δ*bipA,* Δ*bipA::bipA,* Δ*t6ss,* or Δ*rovC* equal to 1 OD_600_ were added to 2 mL PMH minimal media ± 1.5 mM H_2_O_2_ (from a 3% solution made fresh from 30% stock [Fisher] and kept in the dark) and spun in a roller drum at 26°C for 3 h. Concentration (via OD_600_) was measured at 3 h, samples were isolated in 1 mL TRIzol reagent (Invitrogen #15596026) for RNA extraction, and 100 µL was plated on BHI for CFU enumeration.

### Generation of Pst recombinant protein and Pst-specific antiserum

The Pst ORF was amplified from WT *Y. pestis* genomic DNA with primers 5′ Pst_NcoI CCATGGGTTCAGATACAATGGTAGTGAATGGTTCAG and 3′ Pst_XhoI CTCGAGTTTTAACAATCCACTATCGATATCTTTTTGCACC. The resulting amplicon was TA-cloned into pGEM-T Easy (Promega), and after sequence confirmation, the Pst ORF was subcloned into pET-28a via NcoI and XhoI restriction sites to produce a Pst fusion with a C-terminal His_6_ tag. To express recombinant Pst-His_6_, C41(DE3) was transformed with pET28::Pst (Lucigen, Middleton, WI, USA). Cultures were induced for 4 h with 1 mM isopropyl-1-β-D-thiogalactopyranoside at 25°C. Recombinant Pst was affinity purified with HisPur nickel-charged nitrilotriacetic acid resin under native conditions (Thermo Scientific, Rockford, IL, USA). Cells were harvested by centrifugation, suspended in 20 mM Tris-HCl (pH 7.5), 20 mM NaCl, 10 mM imidazole, and 5% glycerol, lysed with BugBuster and Lysonase (Millipore Sigma, Burlington, MA, USA), and clarified by centrifugation. After incubation of the lysate with HisPur resin, the resin was washed sequentially with 20 mM Tris-HCl (pH 7.5), 20 mM NaCl, 20 mM imidazole, and 5% glycerol and 20 mM Tris-HCl (pH 7.5), 1 M NaCl, and 5% glycerol. Protein was eluted with 20 mM Tris-HCl (pH 7.5), 200 mM NaCl, 250 mM imidazole, and 5% glycerol. Fractions containing recombinant Pst were combined and further purified on a HiPrep 16/60 Sephacryl S-200 HR gel filtration column using an Äkta pure 25 L1 fast-performance liquid chromatography system (Cytiva, Marlborough, MA, USA). The recombinant protein was resolved in 20 mM Tris-HCl (pH 8.0) and 150 mM NaCl, and samples of the fractions were analyzed by sodium dodecyl sulfate-polyacrylamide gel electrophoresis (SDS-PAGE) to assess yield and purity. Fractions containing recombinant Pst were pooled and concentrated with an Amicon Ultra-15 30 kDa molecular weight cutoff centrifugal filtration unit (EMD Millipore). Pst-specific antiserum was generated in rats as previously described with minor modifications (95). Briefly, 60 µg of recombinant Pst was electrophoresed by SDS-PAGE, stained with non-fixing Coomassie blue, and gel slices containing Pst were excised. Gel slices were pulverized in a disposable mortar and pestle with sterile phosphate-buffered saline and combined with an equal volume of AddaVax adjuvant (InvivoGen, San Diego, CA, USA). Three- to four-week-old, female Sprague-Dawley rats (Charles River Laboratories, Wilmington, MA, USA) were injected subcutaneously at two sites with 200 µL of emulsion per injection (total of ~25 µg of recombinant protein). Rats were boosted twice at 4-week intervals with ~25 µg of recombinant protein and AddaVax. Rats were euthanized, and serum was collected 2 weeks after the second boost.

### Western blots

To obtain protein extracts from broth-grown *Y. pestis*, overnight cultures of *Y. pestis* CO92 *pgm*−, *Y. pestis* CO92 *pgm*− Δ*bipA,* or Δ*pst* cultures in BHI were subcultured in 10 mL PMH minimal media for 3 h before being spun down at 10,000 × *g* for 10 min to pellet bacterial cells. The pellet was resuspended in 1× B-PER Bacterial Protein Extraction Reagent (Thermo Scientific #78243) + 2 µL 0.5 M EDTA and incubated at 96°C for 3 min to ensure complete lysis. Equal concentrations (approximately 20 ng) of each sample were mixed with the appropriate volume of 6× protein loading dye and run on a 12.5% Tris-glycine pre-cast gel (Bio-Rad #4561045). The blot was transferred to a nitrocellulose membrane with the Bio-Rad TurboBlot Transfer machine and the “Mixed Molecular Weight” protocol. The membrane was washed in 1× TBS before incubating in 3% nonfat milk (Great Value) blocking solution for 1 h. Next, the membrane was rinsed with 1× TBST, followed by overnight cold room incubation in blocking solution containing 1:1,000 α-Pst rat sera. The following morning, the membrane was washed with 1× TBST before the addition of the secondary antibody (1:10,000 goat α-rat) and 1:5,000 Precision Protein StrepTactin HRP Conjugate (Bio-Rad #1610381), followed by incubation for 1 h at room temperature. The membrane was washed with TBST and TBS before developing with the Bio-Rad Clarity Western ECL kit. Images were captured with ChemiDoc. As a protein loading control, nitrocellulose membranes were stripped following the Abcam mild stripping buffer protocol and re-probed with 1:1,000 rabbit α-GroEL (Abcam #ab90522) followed by goat α-rabbit + HRP Conjugate as before.

### Densitometry

Raw JPEG files from ChemiDoc were uploaded to ImageJ for relative quantification of Western bands via pixel quantification method as previously described (96).

### Quantitative reverse transcription PCR

A volume of 2 mL of overnight cultures in BHI or 3 h subcultures in BHI or PMH was spun down at 10,000 × *g* for 3 min to pellet bacterial cells before resuspension and lysis in 1 mL TRIzol reagent (Invitrogen #15596026). Total RNA was then isolated from the cultures per the manufacturer’s instructions and treated with DNase I using the TURBO DNA-*free* kit (Invitrogen #AM1907). Then, 1 µg of RNA from each sample was reverse transcribed to generate cDNA using the Superscript IV polymerase kit (Invitrogen #18090050). Real-time quantitative reverse transcription PCR was performed using PowerUp SYBR green master mix (Applied Biosystems #A25776) with a QuantStudio 6 system, and fold changes were calculated using the delta delta cycle threshold (ΔΔ*C*_*T*_) method normalized to the expression of the housekeeping gene DNA gyrase subunit B (gyrB) from the same sample. Fold changes are represented relative to the expression in the background strain (i.e., *pgm*− or *pCD1*−).

### Statistical analyses

Statistical analyses were done using one-way and two-way analysis of variance and Student’s *t*-test where applicable. *P < 0.05 , **P < 0.005 , ***P < 0.0005, and **** *P* < 0.0001. Unless otherwise stated, all experiments represent three or more replicates. All statistical analyses were performed using GraphPad Prism version 7.04 or later software.
